# Métastase pleurale et pulmonaire d’une polyadénofibromatose dégénéréé: à propos d’un cas

**DOI:** 10.11604/pamj.2017.28.115.13599

**Published:** 2017-10-06

**Authors:** Kawtar El Hachimi, Hanane Benjelloun, Nahid Zaghba, Najiba Yassine

**Affiliations:** 1 Service des Maladies Respiratoires, CHU Ibn Rochd, Casablanca, Maroc

**Keywords:** Adénofibrome, adénofibromatose, pleurésie, métastase, Adenofibroma, adenofibromatosis, pleurisy, metastasis

## Abstract

La polyadénofibromatose ou l’adénofibromatose est définie par la présence d’au moins 3 adénofibromes, uni ou bilatéraux atteignant une taille importante responsable de troubles trophiques. Nous rapportons une observation colligée au service des maladies respiratoires du Centre Hospitalier Universitaire Ibn Rochd de Casablanca. Il s’agit d’une patiente âgée de 46ans, suivie depuis l’âge de 30 ans pour une adénofibromatose bilatérale opérée à 4 reprises. Suite à un bilan préopératoire d’une mastectomie bilatérale, une radio du thorax a été réalisée objectivant un hémithorax droit opaque avec refoulement des éléments du médiastin. L’examen clinique retrouvait un syndrome d’épanchement liquidien de l’hémithorax droit et une adénopathie cervicale sus claviculaire gauche. La ponction biopsie pleurale confirmait la localisation pleurale d’un carcinome peu différencié et invasif compatible avec une origine mammaire. La bronchoscopie après évacuation pleurale objectivait un aspect infiltré de tout l’arbre bronchique, dont les biopsies concluaient au même résultat anatomopathologique. Le traitement préconisé était une polychimiothérapie. L’évolution était marquée par l’apparition de métastases hépatiques. A travers cette observation, nous concluons que les adénofibromes nécessitent une surveillance régulière vu le risque de dégénérescence vers le cancer du sein qui est une cause fréquente de métastases pleuropulmonaires.

## Abstract (en)

Multiple adenofibromas or adenofibromatosis is characterized by the presence of at least 3 mono- or bilateral adenofibromas increasing significantly in size, causing trophic disorders. We report the case of a 46-year old female patient who had been followed up since she was 30 years old for bilateral adenofibromatosis that required 4 surgical procedures. Patient’s medical data were collected in the Department of Respiratory Diseases at the Ibn Rochd University Hospital Center, Casablanca. Basing on preoperative assessment before bilateral mastectomy, the patient underwent chest X-ray that showed opaque right hemithorax exerting compression on the mediastinum. Clinical examination showed effusion syndrome in the right hemithorax and left supraclavicular cervical adenopathy. Pleural puncture biopsy confirmed the presence of poorly differentiated invasive carcinoma in the pleura, supporting breast origin. Bronchoscopy after pleural puncture objectified infiltration of the whole bronchial tree; biopsies confirmed the anatomopathological results. The recommended treatment strategy was based on multidrug chemotherapy. Patient’s evolution was marked by the occurrence of hepatic metastases. This study shows that adenofibromas require regular monitoring given the risk of trasformation to breast cancer, which is a frequent cause of pleuropulmonary metastases.

## Introduction

Les fibroadénomes ou adénofibromes (AF) sont des lésions bénignes le plus souvent rencontrées chez l’adolescente et la jeune femme de la deuxième et la troisième décennie [1]. L’adénofibrome est l’une des principales lésions épithéliales bénignes sans risque carcinologique. Ce dernier pourrait correspondre au risque de dégénérescence révélant une lésion maligne [2]. Leur taille est généralement inférieure à 3 centimètres [2]. Adénofibromatose ou maladie poly-adénofibromatose est définie par des adénofibromes multiples qui s’observent dans 10 à 20% des cas, de façon synchrone ou successive, particulièrement chez les jeunes filles de race noire [3]. Les métastases pleuro-pulmonaires des cancers du sein sont fréquentes. Elles viennent en 3^ème^position après les métastases ganglionnaires et hépatiques et ils sont retrouvés dans 30% des autopsies de patients porteurs d´une néoplasie [4].

## Patient et observation

Il s’agit d’une femme âgée 46 ans, troisième pare, suivie pour adénofibromatose bilatérale ou polyadénofibromatose depuis l’âge de 30 ans, opérée à quatre reprises pour des adénofibromes géants en raison d’une gêne esthétique et fonctionnelle. La patiente est adressée en consultation de pneumologie pour un épanchement pleural liquidien droit découvert fortuitement lors d’un bilan pré-opératoire pour une éventuelle mastectomie bilatérale.

**Figure 1:**
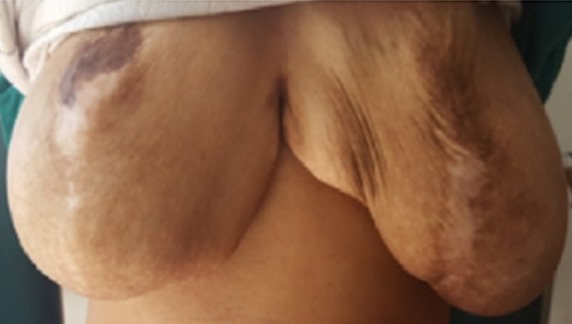
Image montrant des seins asymétriques (vue de face)

**Figure 2:**
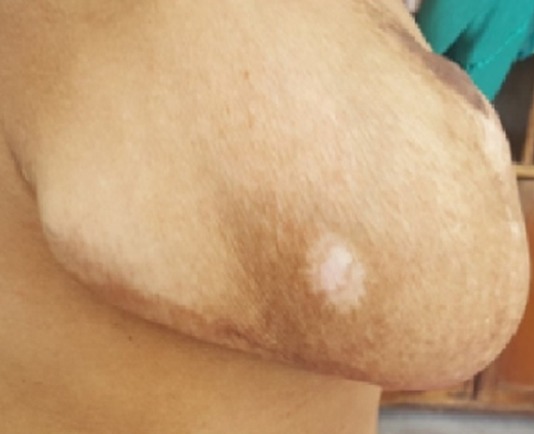
Image montrant des seins asymétriques (vue de profil)

**Figure 3:**
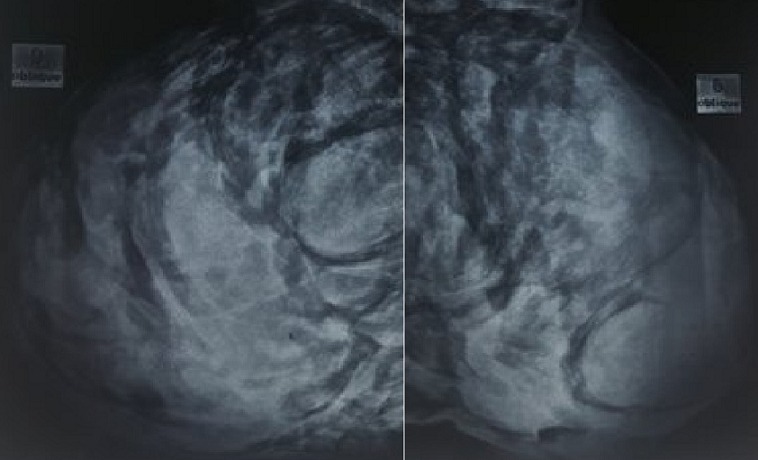
Mammographie montrant de multiples masses au niveau du sein

**Figure 4:**
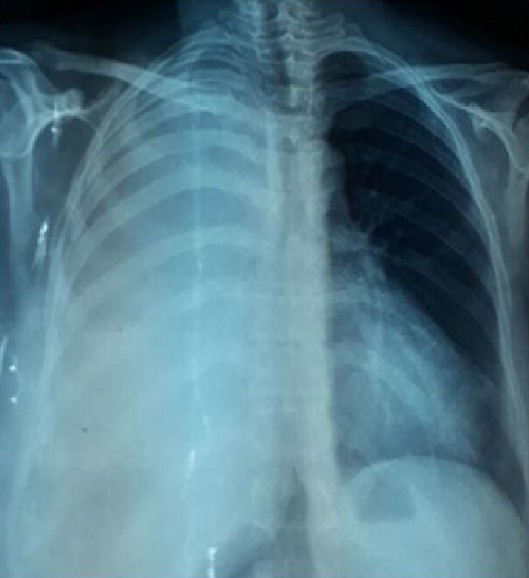
Radio du thorax face montrant un hémithorax droit opaque avec refoulement des éléments du médiastin

L’examen du thorax révélait un syndrome d’épanchement liquidien droit. L’examen mammaire révélait des seins très augmentés de volume, asymétriques, sièges de masses de tailles différentes, non douleureuses, sans signes inflammatoires en regard, ainsi que trois cicatrices propres de chirurgie des adénofibromes (Figure 1, Figure 2). Le reste de l’examen somatique trouvait une adénopathie sus claviculaire gauche infracentimétrique, sans signes inflammatoires en regard, reconnue par la patiente depuis un mois. La mammographie a montré aussi des multiples masses de tailles différentes (Figure 3). A la radiographie du thorax, on a noté un hémithorax droit opaque avec refoulement des éléments du médiastin (Figure 4). Le complément d’imagerie (TDM thoracique) objectivait une pleurésie droite de grande abondance. A la ponction pleurale, la plèvre était fine et le liquide était sérohématique. La ponction biopsie pleurale était en faveur d’une localisation pleurale d’un carcinome peu différencié et invasif compatible avec une origine mammaire. Les ponctions pleurales évacuatrices ont totalisé deux litres de liquide sérohématique. Une bronchoscopie faite après évacuation pleurale a montré une infiltration tumorale de tout l’arbre bronchique droit réduisant le calibre de tous les orifices devenus incathétérisables.

**Figure 5:**
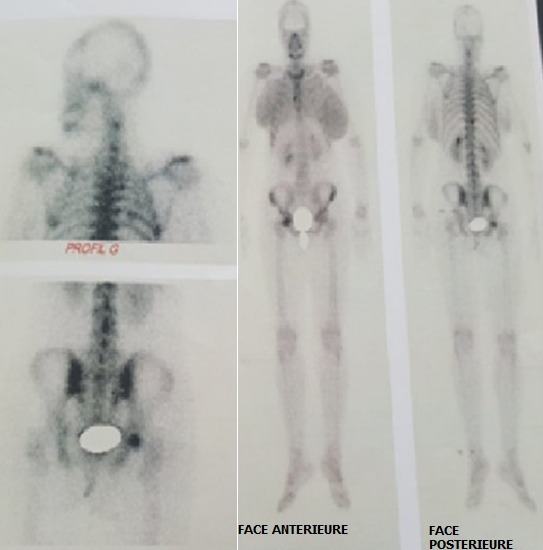
Scintigraphie osseuse qui montre des foyers de localisations osseuses secondaires

Les résultats des biopsies bronchiques concordaient avec ceux de la ponction biopsie pleurale. Il s’agit d’un carcinome peu différencié et invasif compatible avec une origine mammaire, dont l’étude immunohistochimique a montré l’absence de surexpression de l’HER2 par les cellules tumorales (HER2 score 0). Dans le cadre du bilan d’extension, la tomodensitométrie (TDM) cérébrale est revenue normale ainsi que la TDM abdomino-pelvienne. Par contre, la scintigraphie osseuse a révélé un foyer d’hyperfixation modérée de C2, un foyer d’hyperfixation assez intense de D9, un foyer d’hyperfixation du bord droit du corps sternal, et un foyer d’hyperfixation intense de la tête humérale droite (Figure 5). Ces foyers étaient en faveur de localisations osseuses secondaires. La décision de la réunion de concertation pluridisciplinaire a été une polychimiothérapie à base de taxanes. Cliniquement après trois cures de chimiothérapies, on a noté une régression des masses mammaires et persistance des adénopathies. La TDM thoraco abdomino-pelvienne de contrôle a montré des infiltrats pulmonaires droits, et une hépatomégalie siège de 8 nodules de taille différente, en faveur de lésions secondaires.

## Discussion

Les adénofibromes (AF) ou fibroadénomes du sein sont des tumeurs bénignes à prolifération intraparenchymateuse à partir des canaux intra-lobulaires et des acinis. Les adénofibromes multiples sont appelés adénofibromatose ou poly-adénofibromatose. Lorsque la taille est supérieure à 5 centimètres avec une croissance rapide, on parle d’adénofibrome géant [5]. L’adénofibrome est soit de type « classique » sur le plan histologique, défini alors par sa taille ou par son poids dépassant 500g, soit de type juvénile s’il possède un stroma particulièrement cellulaire, caractéristique à cet âge [6, 7]. Il n’existe pas de données d’incidence des adénofibromes dans la population générale. Les AF représentent environ 50% de toutes les biopsies du sein. Ce taux arrive à 75% pour les biopsies chez les femmes de moins de 20 ans [8, 9].

Dans la poly-adénofibromatose, les masses mammaires peuvent entrainer une gêne esthétique et fonctionnelle comme nous l’avons constaté dans notre observation. Les mécanismes à la base de la genèse et de la croissance des fibroadénomes sont peu connus [2, 10]. En pratique, il peut être difficile devant la multiplicité des nodules, de les comptabiliser [3]. Le risque relatif de développer un cancer pour une jeune femme porteuse de fibroadénome sans antécédent familial n’est pas augmenté [2]. La transformation maligne de la composante épithéliale des lésions d’adénofibromes est considérée comme rare. L’incidence rapportée varie de 0,002% à 0,0125% [8]. Certains auteurs rapportent une coexistence de l’adénofibrome avec des carcinomes lobulaires in situ, des carcinomes lobulaires infiltrants, des carcinomes canalaires in situ et des carcinomes canalaires invasifs. L’incidence de cette coexistence varie de 2/1000 à 1,25/1000 dans les adénofibromes prouvés histologiquement [11, 12]. En cas de diagnostic d’adénofibrome à la biopsie percutanée, avec une discordance radioclinique, ou associant des lésions complexes ou prolifératives ou un antécédent familial de cancer, il semble légitime de discuter la prise en charge en réunion de concertation multidisciplinaire et une surveillance est nécessaire afin d’éviter les interventions itératives. Les modalités de prise en charge thérapeutique sont un traitement d’exérèse ou destructeur, un traitement par exérèse percutanée guidée par imagerie ou un traitement médical à base du Tamoxifène, à la posologie de 20mg/jour [4, 13]. Quand l’augmentation de la taille est rapide, le National Cancer Institute recommande une biopsie interventionnelle ou une exérèse chirurgicale [14]. Si l’exérèse chirurgicale est décidée pour un adénofibrome, les incisions directes peuvent être privilégiées afin d’obtenir un meilleur résultat esthétique. L’abord axillaire est associé à un résultat esthétique satisfaisant et une grande satisfaction des patientes selon une étude rétrospective portant sur 50 patientes [15]. L’abord sous mammaire est associé à la persistance d’une asymétrie mammaire post opératoire [7], comme nous l’avons constaté dans notre observation.

En cas d’un nodule gênant de 4 à 5 centimètres, ou plus une exérèse chirurgicale sera pratiquée, dans la crainte d’une tumeur phyllode du sein (TPS) qui est une tumeur mammaire rare associant une double composante lésionnelle fibro-épithéliale. Ces lésions représentent moins de 0,5% de l’ensemble des tumeurs du sein et seulement 2,5% des tumeurs fibro-épithéliales. Selon l’organisation mondiale de la santé (OMS) proposée en 1981, il est usuel de distinguer trois catégories de TPS: grade 1 (bénin), grade 2 (borderline), grade 3 (malin) [16]. A l’inverse, on peut observer une diminution de la taille significativement plus fréquente chez les jeunes filles de moins de 20 ans, que l’adénofibrome soit unique ou multiple, inférieur ou supérieur à 2 cm [3].

Les métastases pleuro-pulmonaires (MPP) ont une prévalence de 30 à 50% chez les patients porteurs de néoplasie thoracique ou extrathoracique [17]. Chez environ 10% des patients qui présentent un épanchement pleural malin, le site primitif n´est pas retrouvé [18]. Le carcinome mammaire est la seconde cause d´épanchement malin (25%) après le cancer du poumon. Les métastases pulmonaires des cancers du sein sont fréquemment retrouvées dans la littérature et surviennent essentiellement par voie hématogène ou lymphatique, elles représentent 20 à 35% des séries [19]. Le développement du foyer pleural initial pourrait s´effectuer à partir d´une extension tumorale directe par contiguïté ou également d´une extension via les lymphatiques ou les vaisseaux sanguins. Aussi, des métastases hépatiques et osseuses peuvent survenir de façon simultanée au cours de néoplasies du sein [4] et que c’est les cancers du sein sans récepteur hormonal qui fabriquent des métastases hépatiques (le cas de notre observation), pulmonaires, cérébrales et méningées.

## Conclusion

Suite à notre observation, nous concluons que l’adénofibromatose nécessite une surveillance étroite dans la crainte d’une dégénérescence maligne menant à un cancer du sein responsable de métastases fréquentes.

## Conflits d’intérêts

Les auteurs ne déclarent aucun conflit d'interêts.
